# Case report of one month and 15 days old baby with type V hyperlipoproteinemia (HLP)

**DOI:** 10.1186/s12902-020-0502-0

**Published:** 2020-02-11

**Authors:** Shabnam Dildar, Tahir Sultan Shamsi

**Affiliations:** Department of Pathology, National Institute of Blood disease and Bone Marrow Transplantation (NIBD), ST 2/A Block 17 Gulshan-e-iqbal KDA Scheme 24, Karachi, 74800 Pakistan

**Keywords:** Type I and V hyperlipoproteinemia, Triglycerides (TG), Refrigerator test, Cholestyramine, Pre-beta lipoproteins, Beta lipoproteins

## Abstract

**Background:**

Most of the patients with type 1 and V hyperlipoproteinemia (HLP) present with symptoms and signs of acute pancreatitis due to marked elevation of triglycerides, but this baby presented with a chest infection, which was later diagnosed as type V HLP on laboratory workup.

**Case presentation:**

We report a case of a 1 month and 15 days old baby boy, product of 2-nd degree consanguinity admitted to a nearby hospital with complaints of refusal to feed, cough and excessive crying. On examination his heart rate was 102 beats/min, respiratory rate was 55 breaths/min and temperature was within the normal range, provisional diagnosis of Pneumonia was made. His samples were tested at our laboratory, the lipid Profile at age of 1 month 15 days showed total cholesterol (TC) of 1400 mg/dl reference range (RR < 200 mg/dl), triglycerides (TG) of > 885 mg/dl after dilution it was 31,400 mg/dl (RR < 150 mg/dl), High density Cholesterol (HDL) of 35 mg/dl (RR > 40 mg/dl) and low density cholesterol (LDL) of 200 mg/dl (RR < 100 mg/dl). The patient’s blood sample was grossly milky and lipemic in appearance. A “Refrigerator test” was performed after overnight storage of the sample in refrigerator at 4 °C, which gave a creamy layer at the top and clear infranatant due to caking of the Chylomicrons. Lipoprotein electrophoresis performed 1 month later showed Chylomicrons of 4.7% (RR 0–2%), Pre-beta lipoproteins of 51.5% (RR 5–22%), beta lipoproteins of 16.5% (RR 39–70%) and alpha of 27.3% (RR 23–53%). Initially he was diagnosed as type 1 HLP, but later on he was correctly diagnosed as type V HLP. Cholestyramine (Questran sachet) powder was started at a dose of 100 mg/kg on t.i.d basis with NAN-1 formula Milk at the age of 1 month and 15 days. On follow up, detailed advices regarding the weaning food was given to the mother (using olive oil in cooking, giving proteins and avoiding heavy fatty meals). His lipid profile was repeated at age of 3 months, which showed some improvement, his TGs were 1986 mg/dl and TC 105 mg/dl.

**Conclusion:**

There is no universal diagnostic criterion for diagnosing Type V HLP, most likely, due to a scanty literature on this disorder. It stimulated us to report this case so that our findings may help for a timely diagnosis of the affected patients.

## Background

Most of the patients with type 1 and V hyperlipoproteinemia (HLP) present with symptoms and signs of acute pancreatitis due to marked elevation of triglycerides levels, but this baby presented with a chest infection instead which was later diagnosed as type V HLP on the laboratory workup.

Lipids, such as cholesterol and triglycerides, are insoluble in plasma and are therefore transported in the plasma via Lipoproteins. Lipoproteins consist of esterified and unesterified cholesterol, triglycerides, phospholipids, and proteins [1]. Lipoproteins are complex molecules consisting of central hydrophobic lipids primarily cholesterol esters and triglycerides surrounded by free cholesterol, phospholipids, and apolipoproteins. Lipoproteins are classified into seven classes based on their size, lipid composition and apolipoproteins; these are Chylomicrons, Chylomicrons remnants, VLDL, IDL, LDL, HDL, and Lpa [2]. The adult treatment panel (ATP) III, National Lipid Association and the National Cholesterol Education Program (NCEP), and the National heart, lung and blood institute of National Institutes of Health (NIH) in 2001 have set the cutoffs for total Cholesterol, with < 200 mg/dl being optimal, 200–239 mg/dl as borderline high, ≥ 240 mg/dl as high. Triglyceride: < 150 mg/dl is optimal, 150–199 mg/dl is borderline high, 200–499 mg/dl is high and ≥ 500 mg/dl is considered as very high. LDL cholesterol should be < 100 mg/dl, level 100–129 mg/dl is above desirable, level is 130–159 mg/dl borderline high, level 160–189 mg/dl is high and ≥ 190 mg/dl is very high. Whereas HDL cholesterol range for males is ≥40 mg/dl and that for females is ≥50 mg/dl [3, 4].

Lipid disorders are of two types, primary and secondary. Primary dyslipidemias occur due to an increase production or decreased removal of lipoproteins; whereas secondary dyslipidemias occur due to an abnormality in either the lipoprotein itself or in the lipoprotein receptors. A well recognized classification system of Fredrickson and World Health Organization (WHO), International Classification of Diseases (ICD) classifies Hyperlipoproteinemia (HLP) phenotypes based on the pattern of the lipoprotein fractions. Fredrickson type V HLP is also called as hyperchylomicronemia late onset, Hyperchylomicronemia with hyperprebetalipoproteinemia, familial Hyperlipemia combined fat and carbohydrate induced hyperlipemia, mixed Hyperlipidemia type V and Type V HLP. Increased Chylomicrons levels are present in Frederickson type 1 and type 5 HLP [5–7]; these gives a milky “latescent” or “lipemic” appearance to the blood sample, due to the presence of increased Chylomicrons (CM) which floats in the blood and give a creamy appearance to the supernatant [8–11]. Type 1 HLP occurs due to the deficiency of extra hepatic lipoprotein lipase (LPL) enzyme or its cofactor apolipoproteins C-II (Apo-II), this insufficiency or deficiency causes marked elevation of TG rich plasma Chylomicrons [12, 13]. Whereas etiology of type V HLP is complex with variable inheritance and it develop in genetically susceptible individuals. Both types (HLP 1 and 5) have elevated Chylomicrons, which gives a creamy top layer to the blood sample. Type V has elevated VLDL Cholesterol with turbid infranatant and the frequency of this disorder is about 5% [14]. Whereas type 1 doesn’t have VLDL elevation, it has clear infranatant and frequency of this disorder is less than 1% [14, 15].

## Case presentation

We report a case of a 1 month 15 days old baby boy, he was admitted to a nearby hospital with the provisional diagnosis of Pneumonia and his sample was sent to our hospital for laboratory testing. The blood sample on gross examination appeared milky, and we therefore took a detailed history from the parents with their consent. He was the product of 2nd-degree consanguinity, first child with no sibling’s, born full term via spontaneous vaginal delivery (SVD) with a normal antenatal history. His developmental history was normal and was up to date on all the immunizations. The past medical and the family history were not significant for Hyperlipidemia.

He was admitted to the emergency ward of a nearby hospital with a history of refusal to feed, cough and excessive cry. On examination his heart rate was 102 beats/min, respiratory rate was 55 breaths/min, temperature was 37 °C and oxygen saturation was 98%.

The laboratory testing revealed the following: Complete blood count (CBC) done on XN-1000 Sysmex hematology analyzer by flow cytometry technique showed hemoglobin of 6.8, White blood cell count of 15.8 and platelets of 161. The sample that was obtained.

appeared milky and lipemic and therefore, he was also screened for Hyperlipidemia. Lipid profile was done by enzymatic method on Cobasc-311 analyzer; its analytical measuring range (AMR) for triglycerides is 8.85 mg/dl to 885 mg/dl. Analyzer gave the result of > 885 mg/dl, since the patients’ physician requested for the exact levels, the sample was rerun, and the results were reported after manual serial dilutions of 1: 50 and multiplication with factor. HDL and LDL levels were performed by the Homogeneous enzymatic colorimetric method on Cobas c-311 analyzer with Analytical Measurement Range (AMR) of 3.09–150 mg/dl and 2–548 mg/dl respectively. Serum amylase and lipase was done on Erba XL-200 by enzymatic method, while liver function tests were done by IFCC kinetic method on the Cobas c-311 analyzer as shown in Table 1.

**Table 1:** Routine laboratory investigation of patient

Routine lab investigations	Results	Reference range
Hb (g/dl)	6.8	9.4–13.0 g/dl
Total Wbc count (X10^9^/L)	15.8	5.00–15.0 X10^9^/L
Platelets (X10^9^/L)	161	210–650 X10^9^/L
ALT or SGPT (U/L)	05	< 45 U/L
ALP (U/L)	477	122–469 U/L
Total Bilirubin (mg/dl)	0.20	0–2.0 mg/dl
Direct Bilirubin (mg/dl)	0.69	0–0.2 mg/dl
Lipase (U/L)	35.8	< 38 U/L
Amylase (U/L)	26	< 100 U/L
HIV 1 and 2	Non-Reactive	Cut off < 1
HepB-sAg	Non-Reactive	Cut off < 1
Anti-HCV Ab	Non-Reactive	Cut off < 1
Syphilis	Non-Reactive	Cut off < 1

**Fig. 1:**
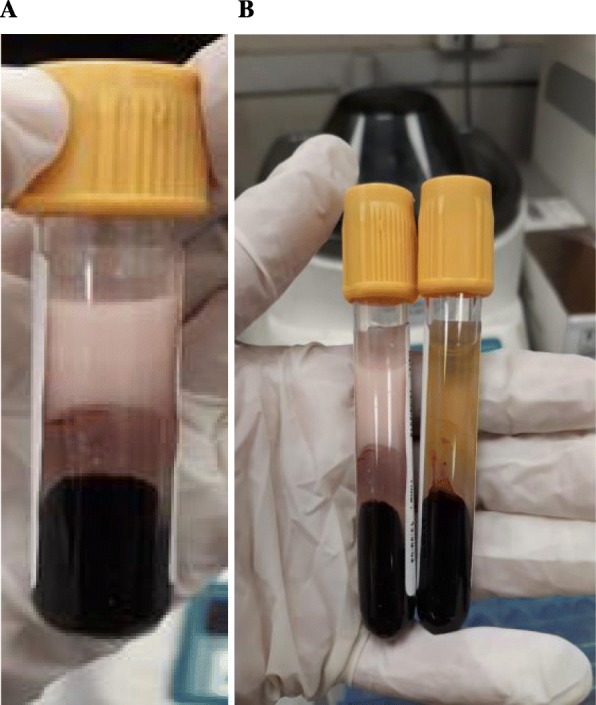
**a** Patient’s sample with creamy supernatant when refrigerated overnight at 4 °C. **b** Patient’s sample along with normal sample

Electrocardiography showed severely hypertrophied normal sized left ventricle with a normal systolic function likely due to non-obstructive hypertrophic cardiomyopathy. Age wise lipid profile is shown in Table 2. A” refrigerator test” was performed after overnight storage of blood sample in the refrigerator at a temperature of 4 °C, which revealed the appearance of creamy supernatant due to the caking of Chylomicrons (shown in Fig. 1). Other tests like Apolipoproteins B (Apo B), ApoA-V, lipoprotein (a) Lp(a), ApoC-11 and Glycosylphosphatidyl inositol anchored high density lipoprotein binding protein 1 (GPIHBP1) could not be done because of unavailability of these tests in our laboratory and the country at large, and patient’s parents were reluctant to send the samples abroad because of financial limitations. Lipid lowering agents were not started as we could not find any recommendations for its use below 3 months of age. Initially he was diagnosed as type 1 HLP, but later at the age of 2 months and 16 days his lipoprotein electrophoresis was done which showed elevated levels of VLDL or Pre-beta lipoproteins 51.5% (RR 5–22%) and Chylomicrons 4.7% (RR 0–2%) shown in Table 3. Cholestyramine (Questran sachet) powder was started at dose of 100 mg/kg on t.i.d basis with NAN 1 formula milk at the age of 1 month and 15 days and on follow up visit detailed weaning food advices were given to the mother (use of olive oil during cooking of weaning food, giving protein and avoiding a diet consisting of fatty meals was advised). His lipid profile was checked at age of 3 months showed TGs 1986 mg/dl (RR < 150 mg/dl) TC was 105 mg/dl (RR < 200 mg/dl), HDL was 5 mg/dl (RR > 40 mg/dl) and LDL was 2 mg/dl (RR < 100 mg/dl) as shown in Table 2.

**Table 2:** Lipid profile at 1 month 15 days, 2 and 3 month

Biochemical investigation	Age of 1 month 15 days	2 months	3 months	Reference range (mg/dl)
Total Cholesterol (mg/dl)	1400	450	105	< 200
Triglycerides (mg/dl)	31,400	2300	1986	< 150
HDL (mg/dl)	35	70	05	< 40
LDL (mg/dl)	200	200	02	< 100

**Table 3:** Lipid electrophoresis at age of 2 month 16 days

Lipoproteins	Result	Reference range
Chylomicrons	4.7	0–2%
Beta lipoproteins	16.5	39–70%
Pre-beta lipoproteins	51.5	5–22%
Alpha Lipoproteins	27.3	23–53%

## Discussion and conclusion

Type V hyperlipoproteinemia (HLP) has many names including hyperchylomicronemia late onset, Hyperchylomicronemia with hyperprebetalipoproteinemia, familial Hyperlipemia combined fat and carbohydrate induced, Hyperlipemia mixed, Hyperlipidemia type V and Hyperlipoproteinemia type 5.

In this case, a ratio of triglycerides to total cholesterol (TG/TC) was 22.4 mg/dl (reference range 5 mg/dl). A ratio of total triglycerides to total cholesterol (TG/TC) above 2.2 (if measured in mmol/l) or above 5 (in mg/dl) is a sign of a high level of circulating Chylomicrons and VLDL. “Refrigerator test” was also performed which yielded a creamy layer at the top and clear infranatant [15]. Lipoprotein electrophoresis showed elevated levels of VLDL or Pre-beta lipoproteins 51.5% (RR 5–22%) and Chylomicrons 4.7% (RR 0–2%) which led to the diagnosis of type V HLP.

Our patient had a very severe hypertriglyceridemia, his TG levels were 31,400 mg/dl and total cholesterol were 1400 mg/dl at the age of 1 month and 15 days; he was started Cholestyramine therapy at that age. His lipid profile was repeated at 3 months of age, which showed TGs reduced to 1986 mg/dl and TC to 105 mg/dl. The endocrinologists have planned to start satins if lipid profile (TGs and VLDL) worsens on the next follow-up.

A case by Manzor et al. in 2015 reported a 10 days old baby girl with symptoms of refusal to feed and irritability. She was a product of 3rd-degree consanguinity, diagnosed based on altered lipid profile which showed total cholesterol of 1236 mg/dl (reference range 170 mg/dl) and triglycerides of 2132 mg/dl. Genetic testing was not done; statins were started along with dietary modification [16].

Pugni et al. in 2014 described the case of a newborn presenting with severe hypertriglyceridemia (> 10 mmol/L), diagnosed with monogenic lipoprotein lipase deficiency, who benefited by exchange transfusion, as a safe alternative to plasmapheresis, in order to prevent acute pancreatitis in young infants with this condition [17].

The type V hyperliproteinemia could be related to genetic factors and acquired or environmental factors (i.e. diabetes, drinking, hormonal therapy, certain drugs, myeloma, SLE, lymphoma and etc). The possible explanation for dyslipidemia development in our case was most likely genetic and not related to environmental factors as the patient was otherwise healthy.

Gotoda T et al. in 2012 stated that environmental factors such as diabetes, drinking hormonal therapy, drugs and myeloma present in only 2/3 of patients with type V hyperlipoproteinemia, while 1/3 patients does not have these factors [18]. The environmental factors were not present in our patient.

This disorder is caused by mutations in different genes such as LPL, Apo B, APOC2, APOA5, GPIHBP1, and LMF1 genes. These mutations are inherited in an autosomal recessive fashion. Our limitation was that we did not perform genetic testing due to non-availability of these tests in the country and due to the financial constraints associated with sending the samples abroad. As per Gotoda T et al., the possibility of LPL deficiency is high if the serum TG level is 1500 mg/dl or higher, the serum total cholesterol level is about 1/10 the serum TG level or lower [18]. Our patient had TG level higher than 1500 mg/dl and his total cholesterol was lower than 1/10. In our case most probable cause of dyslipidemia development is genetic, because he was diagnosed at very young age (1 month and 15 days) there was no any environmental factor involved, furthermore his TC was lower than 1/10 of serum TG, which gives the possibility of LPL deficiency. The Endocrine Society does not recommend routine measurements of these tests for diagnosis of type HLP [19].

A case by Cristina Oana et al. in November 2017 reported a 16-year-old female teenager presenting with severe abdominal pain prominently in the lateral left quadrant, nausea, vomiting and constipation for 2 days. On examination, she had ascites and paralytic ileus. She also had increased level of triglycerides 555.1 mg/dl with normal total cholesterol of 180.5 mg/dl. Lipoprotein electrophoresis showed low level of alpha-lipoproteins (7.6%), increased pre-beta lipoproteins (71.2%), lack of beta-lipoproteins and raised Chylomicrons (21.2%). They found low level of Apolipoproteins A (0.82 g/L), and a normal level of apolipoproteins B. She was diagnosed with type V hyperlipoproteinemia based on lipoprotein electrophoresis and Apo A [15].

In conclusion the literature and case reports on type v hyperlipoproteinemia is scarce. Bianshly Rivera et al. in May 2019 stated that there are no specific diagnostic and management guidelines for this disorder due to insufficient number of cases [20]. Therefore, it prompted us to report this case so that our findings could result in timely diagnosis, treatment and dietary modification and help the affected patients live a normal life.

## Data Availability

All relevant data are included in the case.

## Abbreviations

FCS: Familial chylomicronemia syndrome
LDL: low density cholesterol
TC: Total cholesterol
TG: Triglycerides
TG/TC: triglycerides to total cholesterol
